# Effect of the COVID-19 Pandemic on the Epidemiology of Pediatric Traumatic Brain Injury in Brazil

**DOI:** 10.7759/cureus.48896

**Published:** 2023-11-16

**Authors:** Leopoldo Mandic Ferreira Furtado, José Aloysio Da Costa Val Filho, Victor Da Silva Pereira, Yasmin S Coimbra, Vitor Hugo R Caldas, Anne R Magalhães, Bruna Athayde S De Carvalho, Saulo G Moreira, Antônio L Teixeira, Aline Silva De Miranda

**Affiliations:** 1 Pediatric Neurosurgery, Vila da Serra Hospital / Oncoclínicas, Nova Lima, BRA; 2 Neurosurgery, Vila da Serra Hospital / Oncoclinicas, Nova Lima, BRA; 3 Department of Neurological Surgery, Pontifícia Universidade Católica de Minas Gerais, Contagem, BRA; 4 Department of Neurological Surgery, Faculdade de Ciências Médicas de Minas Gerais, Belo Horizonte, BRA; 5 Department of Neurological Surgery, João XXIII Hospital / Fundação Hospitalar do Estado de Minas Gerais (FHEMIG), Belo Horizonte, BRA; 6 Neuropsychiatry, The University of Texas Health Science Center at Houston, Houston, USA; 7 Department of Morphology, Federal University of Minas Gerais, Belo Horizonte, BRA

**Keywords:** pediatric brain injury, pediatric head trauma, pandemic, covid-19, social distancing

## Abstract

Aim

In response to the coronavirus 2019 disease (COVID-19) pandemic, governments worldwide implemented measures to prevent infection, resulting in restricted school activities, restricted children’s freedom of movement, and increased risk of violence and injuries at home, including traumatic brain injury (TBI), among children. In Brazil, the consequences of the COVID-19 pandemic on the causes, severity, and mortality of pediatric TBI have not yet been investigated. Thus, our study aimed to determine whether the COVID-19 pandemic has affected the epidemiology of pediatric TBI among Brazilian children.

Materials and methods

We investigated the patients with TBI aged <18 years who visited a tertiary trauma center in Brazil in 2019 and 2020. TBI-related variables, such as classification, mechanism, clinical manifestations, need for intervention, morbidity, and mortality, were recorded. Furthermore, we used a nationwide databank to collect information on mortality from external causes of trauma and violence in the pediatric population in 2019 and 2020. The Mann-Whitney test was used to compare quantitative variables related to the mechanisms and severity of TBI in both periods in order to determine the impact of the COVID-19 pandemic.

Results

Of the patients with traumatic brain injury, 1371 visited the trauma center in 2019 and 1052 in 2020. No difference was noted in the incidence rate of abusive head trauma between these periods (p=0.142) or in mortality from violence in Brazil. Recreational causes of pediatric TBI increased during the first year of the COVID-19 pandemic in Brazil and falls from bicycles significantly increased during the pandemic (p<0.001).

Conclusion

A global reduction in pediatric admissions to emergency rooms as well as no impact on mortality and severity of pediatric TBI were observed during the COVID-19 pandemic in Brazil. Additionally, a public education program regarding child safety during recreational activities, particularly how to avoid falls from bicycles was recommended.

## Introduction

The World Health Organization declared the COVID-19 pandemic on March 11, 2020 [1]. The pandemic forced governments globally to take measures to halt the spread of the disease. In Brazil, the first case of COVID-19 was reported on February 26, 2020, in São Paulo, and stay-at-home measures were implemented in the 27 states of Brazil [2].

These measures were supported by the city administration which was responsible for deciding the level of population mobility and were reinforced by the Supreme Federal Court [3]. Minas Gerais has the second highest population (21,411,923) and its capital, Belo Horizonte (population of 2,530,701), was considered a model of social isolation.

The lockdown measures implemented on March 18 included closing schools and malls and restricting sales activities, which helped in controlling the spread of the disease [4]. COVID-19 lockdown measures could lead to economic and social consequences such as loss of employment and an increase in violence [5,6]. In this regard, the risk of violence in families was reported to increase with the incidence rate of inflicted injury owing to economic stress, resulting in the risk of abusive head trauma increasing by 14% for each day of social isolation after the start of the COVID-19 pandemic [1,7-12]. Indeed, this tendency has been reported by previous observations of increased interpersonal violence during natural disasters and unemployment that accompanied economic recession [13-15]. Children were especially affected with regard to their interpersonal relationships: their school activities were restricted and their risk of suffering violence in the home increased [12].

Pediatric traumatic brain injury (TBI) is a global public health concern that affects at least 3 million children every year and leads to an estimated 300,000 pediatric deaths [16]. Few studies in the literature discussed the impact of the pandemic on the epidemiology of pediatric TBI and some discrepancies were observed. In the USA, a retrospective study was conducted in Texas and displayed a significant increase in hospital admissions due to pediatric TBI during the pandemic period, and no differences were observed in the mechanisms of trauma [17]. Differently, in Brazil, Souza et al. performed an epidemiological study among children considering the data obtained from DATASUS, a governmental databank that continually receives information about mortality from several causes in the country, and found the mortality rate of 0.47 per 100,000 pediatric inhabitants per year with the cost of 417.88USD per admission pointed out decrease of admissions during the pandemic [18]. In spite of this, no information regarding the details of pediatric TBI such as mechanisms of trauma was possible due to the limitations of this databank [19]. Therein, this data is unknown so far in Brazil.

Given the socioeconomic impact of COVID-19 in Brazil, including economic recession and unemployment [20], we hypothesized that during the COVID-19 pandemic, the mortality rate due to external causes of trauma, as well as trauma caused by violence, would increase in Brazil, and that referral trauma hospitals would experience an increase in the severity of pediatric TBIs.

Thus, our study aimed to investigate the effect of the COVID-19 pandemic on sociodemographic characteristics, the causes, severity, and mortality rates of pediatric TBI in a referral trauma center in Minas Gerais, Brazil.

This article was previously posted to the ResearchGate preprint server on August 28, 2023.

## Materials and methods

Study design and population

This retrospective study was based on the data obtained from a major trauma center, Hospital João XXIII, which is a public teaching hospital of Belo Horizonte, and belongs to the state hospital foundation. It is a referral hospital for emergencies that attends an average of one thousand pediatric patients with TBI per year [21]. Furthermore, we obtained data from DATASUS( https://datasus.saude.gov.br/) about mortality in Brazil resulting from external causes, such as trauma and violence. This study was approved by the ethics committee of the Hospital of João XXIII (#4.312.892).

Inclusion and exclusion criteria

We included data from all patients younger than 18 years of age who visited Hospital João XXIII and received a diagnosis of TBI. We analyzed data from the period March to October 2019 (before the COVID-19 pandemic) and from the same period of 2020 (during the pandemic). Patients who visited the hospital in 2019 were designated as group 1, and those who visited in 2020 were designated as group 2. Such data were collected in the hospital repository from July to November 2022 after approval by the ethical committee. The patients are identified by numerical codes in order to avoid name identification on the data bank.

Pediatric TBI was defined as any injury caused by an external force to the head resulting in an anatomical lesion or functional impairment of the cranial or encephalic structures in individuals younger than 18 years who presented in emergency care [22]. In order to enhance the accuracy of this study to identify the TBI cases, those patients who suffered solely facial trauma with no neurologic abnormalities were excluded from this study, as were others who suffered from trauma in other body parts but had no injuries of the neurocranium. We also excluded patients whose charts contained no basic information about the mechanism of trauma, classification of TBI, or outcomes.

Variables

Demographic features, such as gender and age, were considered variables in this study. For all patients, the severity of TBIs was classified on the Glasgow Coma Scale as mild (score of 13-15), moderate (score of 9-12), or severe (score of <9) [23]. The mechanism of trauma was classified as types of falls, vehicle accidents, objects by which patients were struck, gunshots, and sports-related injuries. The unequivocal diagnosis of abusive head trauma was based on the child’s entire history of inflicted injury and clinical presentation with signs of violence that were reinforced by the detection of retinal hemorrhage by an ophthalmologist documented in the patient chart [24]. Moreover, other variables, such as periorbital hematoma and anisocoria, headache, severe vomiting or vomiting more than three times, seizures, loss of consciousness, and post-traumatic amnesia were adopted. We also documented whether computed tomographic (CT) imaging of the head was performed, findings such as skull fractures and types of intracranial hemorrhage, the need for neurosurgical intervention, and the length of hospitalization.

The primary outcome was the TBI severity and mortality, which was calculated considering the number of obits per total patients attended on the hospital databank and in the DATASUS databank; secondary outcomes included its causes and mechanisms.

To compare rates of mortality from violence and external causes of trauma, we obtained data about mortality from several causes, including TBI, from DATASUS [18]. In this databank, external causes of trauma included unintentional trauma and violence, but the dataset did not distinguish violence resulting in TBI from other causes of injury. Nonetheless, we were able to compare the overall trend of these statistics in Brazil with that in our single institution and verify whether the institution’s trend reflected the national trend.

Another limitation of this databank is that it was not possible to obtain samples under the age of 18 years due to the data filtering available and the groups of age considered were the following: < 1 year, 1-4 years, 5-9 years, 10-14 years, and 15-19 years.

Statistical analysis

We used SPSS Statistics 20 (IBM Corporation, Armonk, NY, USA), Minitab 16 (Minitab LLC, State College, PA, USA), and Excel Office 2010 (Microsoft Corporation, Redmond, WA, USA) for statistical analysis in this study in which continuous and categorical variables were presented.

To compare the quantitative data of the two groups of patients, with continuous variables, we applied the Mann-Whitney U test, which was used to compare quantitative variables related to mechanisms and severity of TBI among both periods in order to determine the impact of the COVID-19 pandemic. We used a significance level of.05 and a 95% confidence interval.

## Results

During the periods March to October 2019 and March to October 2020, 2423 pediatric patients received emergency care for TBI: 1371 before and 1052 during the COVID-19 pandemic (groups 1 and 2, respectively), representing a 23% decrease in the hospital admission of children with TBI. Concerning sociodemographic features, a mean age of 71.6 months (16.6-225) (SD=66.8) was observed in group 1, and in group 2, the mean age was 69.5 months (16.8-224) (SD=55.9) (p=0.639). No significant difference was observed between the groups regarding pre-verbal age (<2 years) (p=0.753). Group 1 was 463 (33.8%) and group 2 was 343 (32.6%). The majority of patients were boys (833 (60.8%); p=0.479). Clinical manifestations are presented in Table 1.

**Table 1:** Clinical Manifestations of Pediatric Traumatic Brain Injury Before and During the COVID-19 Pandemic

	2019 (before pandemic), n (%)	2020 (during pandemic), n (%)	P value
Headache	126 (9.2)	85 (8.1)	.337
Vomiting	320 (23.3)	245 (23.3)	.976
Seizure after trauma	25 (1.8)	28 (2.7)	.161
Loss of consciousness	234 (17.1)	208 (19.8)	.083
Amnesia	41 (3.0)	23 (2.2)	.220
Signs of basilar skull fracture	42 (3.1)	41 (3.9)	.261
Anisocoria	6 (0.4)	2 (0.2)	.293

Most patients in both groups presented with mild TBI, and the numbers did not differ significantly: 1332 (97.15%) in group 1 and 1007 (95.7%) in group 2 (p=0.106). Moderate TBI was observed in 17 patients (1.2%) in group 1 and 15 (1.4%) in group 2 (p=0.106 ), and severe TBI was observed in 22 (1.6%) in group 1 and 30 ( 2.8% ) in group 2; these differences were also not significant (p=0.100). Although we found no differences between groups 1 and 2 in the incidence of severe pediatric TBI or in clinical presentation, a significant increase in skull fractures and subarachnoid hemorrhage was noted in 2020. The imaging findings and outcomes are listed in Table 2.

**Table 2:** Imaging Findings and Outcomes of Pediatric TBI Before and During the COVID-19 Pandemic

	2019 (before pandemic), n (%)	2020 (during pandemic), n (%)	P value
Imaning findings			
Skull fracture	109 (8.0)	136 (12.9)	<.001
Intracranial hemorrhage	24 (1.8)	19 (1.8)	.918
Epidural hematoma	33 (2.4)	22 (2.1)	.607
Subdural hematoma	27 (2.0)	18 (1.7)	.641
Subarachnoid hemorrhage	19 (1.4)	29 (2.8)	.016
Intraparenchymal hemorrhage	23 (1.8)	23 (2.2)	.361
Ventricular hemorrhage	4 (0.3)	6 (0.6)	.289
Outcomes			
Neurosurgical interventions	18 (1.3)	23 (2.2)	.099
Decompressive craniectomy	3 (0.2)	5 (0.005)	.276
Cerebrospinal fluid leakage	2 (0.1)	0 (0)	.215
Meningitis	2 (0.1)	4 (0.004)	.250
Mortality	7 (0.5)	5 (0.4)	.902

The number in June 2020, during the first relaxation of restrictions, did not differ from that in June 2019, and the number in July 2020, during the second period of social isolation, did not differ from that in April 2020, during the first isolation period. On the other hand, with the second relaxation of restrictions, the numbers in both groups were similar. Of interest was that, despite the second relaxation of restrictions, more severe TBIs occurred during August and September 2020 than in the same period in 2019, mainly as a result of recreational mechanisms of trauma (Table 3).

**Table 3:** Mechanisms of Trauma Before and During the COVID-19 Pandemic

Mechanism	2019 (before pandemic), n (%)	2020 (during pandemic), n (%)	P value
Falls			
To ground	403 (29.4)	239 (22.7)	< .001>
Bed	209 (15.2)	150 (14.3)	.505
Downstairs	125 (9.1)	140 (13.3)	.001
Bicycle	71 (5.2)	94 (8.9)	< .001>
Couch	50 (3.6)	47 (4.5)	.305
Baby drop	44 (3.2)	33 (3.1)	.923
Roof	13 (0.9)	11 (1.0)	.809
Slide	13 (0.9)	1 (0.1)	.006
Horse	5 (0.4)	10 (1.0)	.068
Hammock	5 (0.4)	10 (1.0)	.068
Vehicle accident			
Bus	6 (0.4)	0 (0)	.032
Car	48 (3.5)	46 (4.4)	.269
Motorcycle	11 (0.8)	14 (1.3)	.201
Struck			
By car	18 (1.3)	22 (2.1)	.135
By motorcycle	26 (1.9)	23 (2.2)	.613
By bicycle	3 (0.2)	2 (0.2)	.878
Struck by an object	101 (7.4)	54 (5.1)	.026
Assault	35 (2.6)	14 (1.3)	.034
Gunshot	3 (0.2)	5 (0.5)	.275
Sports-related injury	9 (0.7)	7 (0.7)	.706

For example, falls from bicycles increased significantly during the pandemic (p<0.001). In addition, the incidence of abusive head trauma did not differ significantly between these two periods (p=0.142), although the number of such cases reduced from 45 (3.3%) to 24 (2.3%).

With regard to head imaging examinations, the performance of CT scans and skull radiography differed significantly between 2019 and 2020. The number of patients receiving CT scans increased from 492 (35.9%) in 2019 to 442 (42%) in 2020 (p=0.002), and the number of patients undergoing skull radiography decreased from 249 (18.2%) in 2019 to 120 (11.4%) in 2020 (p<0.001). Furthermore, the mean length of hospitalization was reduced significantly (p=0.003) from 1.09 days in 2019 to 0.83 days in 2020.

We also found no significant difference in rates of mortality from external causes and violence, either at the hospital or in the national databank, between 2019 and 2020 (Tables 3-5).

**Table 4:** Mortality From External Causes in Brazil Before and During the COVID-19 Pandemic From the DATASUS Databank

Age	2019 (before pandemic), n (%)	2020 (during pandemic), n (%)	P value
<1 year	981 (6.0)	863 (5.4)	.015
1–4 years	1209 (7.4)	1213 (7.6)	.590
5–9 years	736 (4.5)	752 (4.7)	.438
10–14 years	1549 (9.5)	1445 (9.0)	.137
15–19	11,835 (72.6)	11,750 (73.3)	.120

**Table 5:** Mortality From Violence in Brazil Before and During the COVID-19 Pandemic From the DATASUS Databank

Age	2019 (before pandemic), n (%)	2020 (during pandemic), n (%)	P value
<1 year	84 (1.2)	72 (1.0)	.297
1–4 years	104 (1.5)	101 (1.5)	.765
5–9 years	69 (1.0)	66 (1.0)	.741
10–14 years	386 (5.7)	349 (5.1)	.116
15–19	6,175 (90.6)	6,314 (91.5)	.062

## Discussion

The present study pointed out that there were no significant differences between the two periods regarding the number of obits due to pediatric TBI and the distribution of the severity of TBI (mild, moderate, and severe) in a hospital reference in trauma as well as in Brazil’s databank expressed by the external causes of trauma and mortality due to violence in the pediatric population. In this regard, the study suggests that the COVID-19 pandemic does not impact the severity of TBI and mortality. Moreover, the study showed a 23.3% reduction in the incidence of the number of admissions of pediatric patients with TBI during the first months of the pandemic compared to the pre-COVID period. Such a finding was supported by another Brazilian epidemiological study in which it was observed that during the pandemic, the incidence of pediatric hospital admissions decreased [18]. This reduction may have occurred as a result of the population’s initial fear of contracting the disease during the first months of the pandemic, leading to avoidance of emergency rooms and hospitals, mainly in mild TBI; this “fear effect” was reported in a study conducted in the United Kingdom [6]. Conversely, recreational causes of TBI such as falls from hammocks and horses have doubled during the pandemic. Probably, this finding could be attributable to the closing of schools and restrictions on children’s social activities, which might have led to exposure to trauma from recreational and potentially dangerous activities during the pandemic. In fact, in Brazil, only 81% of households have access to the Internet; thus, access to remote learning programs is limited for many children, particularly those in rural communities [25].

Another significant finding was that head trauma caused by accidents was more common than TBI from extremely violent mechanisms of trauma such as assault, whose incidence was reduced in 2020, and gunshots, whose incidence did not increase significantly. Interestingly, such a finding is divergent from a cross-sectional study conducted in Atlanta (USA) in which an increase of 10.4% in firearms injuries on the head and neck was observed during the first five months of the pandemic [5]. Furthermore, no significant difference was observed in morbidity such as infection of the central nervous system during the pandemic in comparison with the period pre-COVID-19. Indeed, meningitis and progression to brain abscess are meaningful complications related to TBI representing a challenging diagnosis in some instances [26].

Notably, trauma from falls increased in 2020 (p<0.001), especially from bicycles, and trauma from bus accidents decreased in 2020, which could reflect changes in children’s recreational behavior and reduction of traffic during periods of social isolation, respectively. Such phenomena regarding falls from bicycles were also reported in a retrospective study conducted in Perth, Australia, which revealed a 42.7% increase in trauma related to bicycles during 2020. However, the number of closed-head injuries and concussions did not change significantly; a total of 9 and 11 cases were reported in 2019 and 2020, respectively [27]. Simultaneously, an increasing reduction in the use of helmets during the practice of bikes and other recreational activities was reported during the pandemic period according to Troy et al. [28].

Although falls from bicycles typically occurred in a recreational context, parents who could no longer work in an office and worked at home may have been unable to monitor children who rode bicycles during the day instead of attending school, which could explain these data. This hypothesis was reinforced by the findings of Lee et al., who conducted a survey of 283 parents during the social isolation phase of the pandemic and found that children were more at risk for spanking during rather than before the pandemic (odds ratio=2.32, p<0.05). Lee et al. also reported that the probability of neglecting children increased by 132% during the pandemic [1].

Another unexpected finding of this study was that there was no increase among children in abusive head trauma or in overall mortality from violence, either in the local data or in the data provided by the Brazilian Government. This finding is consistent with the results of Caron et al.’s retrospective French nationwide study, which showed that the rate of mortality from abusive head trauma was 0.016% in 2020 and 0.017% in 2019 (p=0.96). Caron et al. hypothesized that the reason for this unexpected finding was that the presence of more family members at home prevented children from being left alone with one perpetrator, which is the most common risk factor for abusive head trauma [29]. In contrast, Sidpra et al. reported a 1493% increase in abusive head trauma during the first month of social isolation in the United Kingdom [10].

Such data in Brazil could have been underestimated because abusive head trauma remains poorly recognized, whether it results in mortality or not; only a minority of children who suffer physical abuse will be identified as such during medical evaluation [23]. The diagnosis of abusive head trauma could be challenging especially during crises such as a pandemic [9]. Therefore, the population and authorities must be alerted to the risk of abusive trauma during such crises, despite the apparent reduction in TBI during the COVID-19 pandemic.

In most cases of head trauma in children in the emergency room during both periods, injuries were mild, and the incidences of severe and moderate brain injuries in 2019 and 2020 did not differ. The frequencies of CT scanning and skull radiography may thus be of concern in view of the risks from radiation and, simultaneously, the risk of overlooking intracranial bleeding [28]. Another remarkable finding of this study was the significant increase in CT scans and the reduction in skull radiography during the pandemic; this could reflect the effect of stress on families and physicians during a child’s emergency room visit, which tended to be abbreviated during the pandemic (p=0.003). In this hospital, the guidelines of the Pediatric Emergency Care Applied Research Network (PECARN) were adopted as a standard of care for mild TBI assessment in 2018; one of the indications for CT scanning is the shared agreement by parents and physicians in some instances [19,29]. On the other hand, our data revealed the influence of the PECARN guidelines in this hospital. In 2019, skull radiographs were obtained in 52.6% of cases of mild TBI; in contrast, the current rate is 11.4%. Of interest is that the rate of CT scanning was 36.4% before the implementation of PECARN guidelines and it decreased to 35.9% before the pandemic; however, it increased to 42% in 2020, which reflects the negative effects of the pandemic despite the implementation of PECARN guidelines [19]. As a result, more cases of subarachnoid hemorrhage were diagnosed in 2020 (p=0.016), although no other types of brain injuries or neurosurgical interventions were noted (Table 1).

This study is the first to focus on the impact of the COVID-19 pandemic on pediatric TBI in Latin America, focusing on Brazilian children. The findings regarding the mechanism of trauma were unexpected such as falls from bicycles. However, this study had several limitations. Due to the retrospective study design, the results are subject to bias because of the method of data collection and the imprecise documentation of patients’ characteristics. Furthermore, due to the exclusion of all trauma exclusively on the face, some violent trauma mechanisms might have been neglected, which could impact the general epidemiology of nonaccidental trauma mechanisms referred to our hospital. Furthermore, our data only reflect the first year of the pandemic, and changes could have occurred during the subsequent waves of infection.

## Conclusions

The present research showed a global reduction in pediatric admissions to emergency rooms as well as no impact on mortality and severity of pediatric TBI during the COVID-19 pandemic. In addition, TBI caused by recreational activities increased in Brazil, as did the use of CT scanning. Therefore, as a recommendation, the emergency room staff should be attentive to recognizing cases of abusive head trauma and should avoid unnecessary CT scanning, especially during public health crises. Our findings also indicate that the population must be educated about the need for children who ride bicycles to use appropriate protection such as wearing helmets.
